# Femoral Neck Non-Union - A Biomechanical Solution with Valgus Sliding Subtrochanteric Osteotomy: A Case Report

**DOI:** 10.5704/MOJ.2007.022

**Published:** 2020-07

**Authors:** KH Teh, JK Ruben, CK Chan, AA Abbas

**Affiliations:** 1 Department of Orthopaedic Surgery, Universiti Putra Malaysia, Serdang, Malaysia; 2 Department of Orthopaedic Surgery, Hospital Serdang, Kajang, Malaysia; 3 Department of Orthopaedic Surgery, Mahkota Medical Centre, Malacca, Malaysia; 4 Department of Orthopaedic Surgery, University of Malaya, Kuala Lumpur, Malaysia

**Keywords:** femoral neck fracture, non-union, valgus sliding subtrochanteric osteotomy

## Abstract

Avascular necrosis and non-union are two most dreaded complications of femoral neck fracture fixations. Hip replacement seems to be a simple solution for this complex problem. However, the long-term efficacy of prosthetic replacement in the young population with higher functional demand is still questionable. Femoral head preserving valgus subtrochanteric osteotomies in properly selected cases have strong support from literature. The conventional technique of valgus subtrochanteric osteotomy involves lateral based wedge resection. Alternatively, a simpler sliding oblique subtrochanteric osteotomy without any wedge removal can also be performed. We hereby describe a successful case of sliding subtrochanteric osteotomy with 135° dynamic hip screw (DHS) plate fixation in treating non-union neck of femur fracture in a young gentleman.

## Introduction

Femoral neck fracture fixation can be complicated with non-union, especially in Pauwels type-3 fracture owing to high shearing force across the fracture site, with reported average incidence of 12% in literature^1^. In non-union, surgical options are mainly divided into head salvage or sacrificing procedures, depending on the viability of the femoral head. Preservation of the femoral head can be achieved via biological or biomechanical methods. Bone grafts (cancellous, cortical, vascularised fibular grafts or muscle pedicle grafts) improve the microenvironment biology. Valgus hip angulation osteotomy improvises the biomechanical load of the hip joint. It will reorient a vertical fracture into a horizontal fracture, thus converting shearing force into compressive force across the fracture site. However, surgeons nowadays tend to favour the easier option of femoral head sacrificing hip arthroplasty. This provides immediate pain relief and early mobilisation, which is the goal of treatment in elderly patients. However, one must remember the goal of treatment in young adults should be focusing on preserving the femoral head due to the mismatch between their high functional demand and prosthetic implants longevity in the long term. Thus, an alternative, less radical approach like valgus hip angulation osteotomy is worth considering in younger people. We report a case of femoral neck non-union in a young adult treated with valgus sliding subtrochanteric osteotomy and the rationale behind it.

## Case Report

**Fig. 1:**
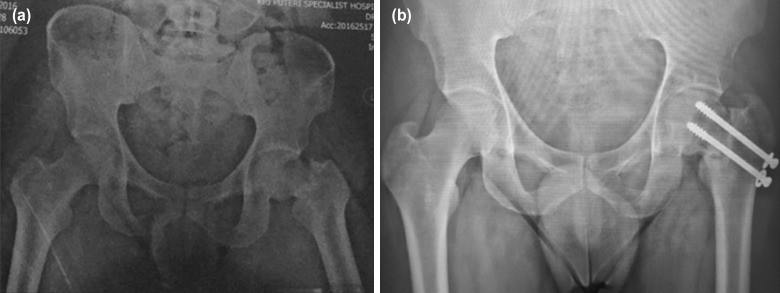
(a) Pelvic radiograph demonstrated left hip basicervical, Pauwels type-3 neck of femur fracture. (b) Pelvic radiograph six months after two cannulated screws fixation, noted non-union over fracture site evidenced by no bridging callus formation and sclerotic fracture edge.

A 33-year-old gentleman suffered a left hip neck of femur fracture from a fall. A pelvic radiograph demonstrated a displaced neck of left femur basicervical fracture, Pauwels type-3. He underwent immediate fracture reduction and fixation with two cannulated screws (Fig. 1a). No immediate post-operative complications were observed.

**Fig. 2:**
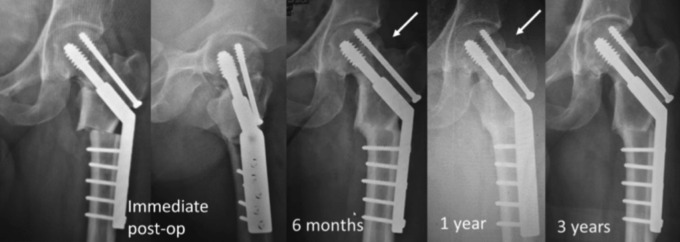
Left hip radiographs after subtrochanteric sliding osteotomy and DHS fixation. Union achieved over osteotomy site (cortical bridging) at six months while femoral neck non-union site (arrow) showed progressive union at three years (fracture line disappearance).

However, six months post-operation, he complained of persistent left groin pain upon moving his left hip, and he was unable to bear weight on the affected limb. Pelvic radiograph (Fig. 1b) showed non-union over fracture site with slight varus collapse, evident by sclerotic edges of the fracture ends. Computer tomography scan of the left hip was done to evaluate the sphericity of the femoral head and the quality of bone stock that were found to be intact. He underwent implant removal, valgus sliding subtrochanteric osteotomy and 135° dynamic hip screw (DHS) fixation. The patient was on partial weight bearing for six weeks and progressed to full weight bearing by twelve weeks. Fig. 2 shows sequential left hip radiographs after the operation. Six months post-operation, union achieved over both femoral neck non-union site and subtrochanteric osteotomy site. Three years after surgery, the patient demonstrated excellent Harris hip score with no limb length discrepancy.

## Discussion

A non-union femoral neck fracture is common because of the absence of periosteal cambium layer and the continuous synovial bathing. These prevent hematoma formation, which in turn leads to decreased healing potential^1^. Thus, obtaining anatomic reduction and stable fixation remains the key to successful treatment of femoral neck fractures. Failure is often a result of not achieving these principles. Initial operation with two cannulated cancellous screws fixation was a suboptimal management for this patient, who had typical Pauwels type-3 basicervical femoral neck fracture. A primary DHS fixation would have provided a more stable fixation. Cannulated cancellous screw fixation is still possible provided three cannulated screws with triangular configuration are used. Three screws offer better stability and can withstand higher load before failure compared to two vertical screws^2^.

**Fig. 3:**
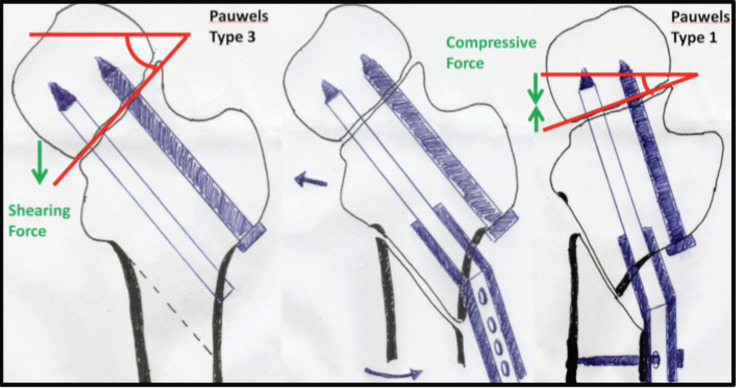
Schematic drawing (from left to right) of valgus sliding subtrochanteric osteotomy and Pauwels angle correction. Dynamic hip screw and derotation screw insertion along the axis of femoral neck, and oblique osteotomy done directed towards the lesser trochanter. The degree of obliquity depends on neck shaft angle. The distal mobile fragment is clamped to barrel plate to achieve the desired correction via lateralisation of the femoral shaft. The Pauwels angle becomes more horizontal after neck shaft angle corrected to barrel plate angle. This technique allows translation only in frontal plane without altering the femoral neck anteversion.

Pauwels classification is more descriptive and useful than Garden classification in describing young adult femoral neck fracture because it predicts the stability of fracture fixation and prognosis^1^. Pauwels type-3 fracture describes femoral neck fracture which is more vertically orientated (more than 50°), resulting in high shearing force across the fracture site that predispose to fixation failure and fracture non-union. Pauwels’ valgus intertrochanteric osteotomy realigns the neck shaft angle; reorient vertical fracture into horizontal fracture pattern. Thus, it converts shearing force to compressive force across the fracture site, creating an optimal healing environment as illustrated in the schematic diagram (Fig. 3). However, there are contraindications to valgus osteotomy such as osteonecrosis with incongruence, poor bone quality and reabsorption. However, radiographic evidence of osteonecrosis alone without collapse is not a contraindication^3^.

Valgus intertrochanteric osteotomy with angle blade plate fixation is a traditional method and a technically demanding procedure^1^. It may displace the fracture fragments due to hammer impact. DHS, which is routinely used for intertrochanteric fractures, provides a technically simple means of fixation for valgus osteotomies in the treatment of femoral neck non-union. The possibility of femoral head rotation can be prevented by derotation screw insertion before DHS reamer. An added advantage over blade plate is DHS provides compression at the fracture site^1^.

Schwartsmann *et al*^3^ reported good outcome treating femoral neck non-union with valgus osteotomy. They have stated their preference of DHS over angle blade plate given DHS provides rigid stability, is less aggressive, easier to perform, and allows compression over non-union site.

Gavaskar *et al*^4^ has described their novel technique of performing sliding subtrochanteric osteotomy over conventional wedge osteotomy. They concluded that sliding osteotomy is simple, does not need extensive pre-operative planning or removal of bone compared to conventional osteotomy. Removing wedges may hinder limb length restoration and requires careful planning and templating to avoid the same. It also increases the surgical time and blood loss. Hence the reasons for choosing this method for fixation in this case study. A precise angle of correction of Pauwels angle is not needed as long as the corrected Pauwels angle is below 50°^5^. This technique of sliding osteotomy minimises problems associated with overcorrection of Pauwels inclination angle occasionally seen in conventional lateral wedge resection which theoretically can lead to avascular necrosis^4, 5^.

We do not recommend this technique as a primary fixation in Pauwels III acute femoral neck fracture because of the potential morbidity associated with the creation of an additional osteotomy. It should only be reserved for management of revision of non-unions and implant failures. Acute fracture fixation still adheres to the principle of anatomic reduction with rigid fixation. Muscle pedicle bone grafting using quadratus femoris muscle or gluteus minimus muscle should be employed if the biological cause of non-union like coexistence avascular necrosis or late presenter is suspected^1^. Occasionally, additional autogenous cancellous bone graft (harvested from ipsilateral greater trochanter) is used to pack the fracture site to further enhance fracture healing.

Treatment of femoral neck non-union in young adults can be challenging with many methods of fixation available. The principle of a revision surgery is to first establish the cause of non-union. The biomechanical problem seems to be the primary cause of non-union detected early. Thus, femoral head preserving surgery should be the mainstay of treatment and valgus sliding subtrochanteric osteotomy provides a viable option.
